# PreC and C Regions of Woodchuck Hepatitis Virus Facilitate Persistent Expression of Surface Antigen of Chimeric WHV-HBV Virus in the Hydrodynamic Injection BALB/c Mouse Model

**DOI:** 10.3390/v9020035

**Published:** 2017-02-21

**Authors:** Weimin Wu, Yan Liu, Yong Lin, Danzhen Pan, Dongliang Yang, Mengji Lu, Yang Xu

**Affiliations:** 1 Department of Pathogen Biology, School of Basic Medicine, Tongji Medical College, Huazhong University of Science and Technology, Wuhan 430030, China; wuweimin2012@hotmail.com (W.W.); liuyan_54321@126.com (Y.L.); linyong1027@163.com (Y.L.); pande20080901@163.com (D.P.); mengji.lu@uni-due.de (M.L.); 2 Department of Infectious Diseases, Union Hospital, Tongji Medical College, Huazhong University of Science and Technology, Wuhan 430022, China; dlyang@hust.edu.cn; 3 Institute of Virology, University Hospital of Essen, 45147 Essen, Germany

**Keywords:** woodchuck hepatitis virus, hepatitis B virus, chimeric genome, mouse model

## Abstract

In the hydrodynamic injection (HI) BALB/c mouse model with the overlength viral genome, we have found that woodchuck hepatitis virus (WHV) could persist for a prolonged period of time (up to 45 weeks), while hepatitis B virus (HBV) was mostly cleared at week four. In this study, we constructed a series of chimeric genomes based on HBV and WHV, in which the individual sequences of a 1.3-fold overlength HBV genome in pBS-HBV1.3 were replaced by their counterparts from WHV. After HI with the WHV-HBV chimeric constructs in BALB/c mice, serum viral antigen, viral DNA (vDNA), and intrahepatic viral antigen expression were analyzed to evaluate the persistence of the chimeric genomes. Interestingly, we found that HI with three chimeric WHV-HBV genomes resulted in persistent antigenemia in mice. All of the persistent chimeric genomes contained the preC region and the part of the C region encoding the N-terminal 1–145 amino acids of the WHV genome. These results indicated that the preC region and the N-terminal part of the C region of the WHV genome may play a role in the persistent antigenemia. The chimeric WHV-HBV genomes were able to stably express viral antigens in the liver and could be further used to express hepadnaviral antigens to study their pathogenic potential.

## 1. Introduction

The hydrodynamic injection (HI) mouse model has been explored in different studies in hepatitis B virus (HBV) research [1]. It has been proven that the HBV mouse model based on HI could be used to study viral replication and persistence in order to analyze the immunological factors required for HBV clearance and to evaluate novel antiviral therapy strategies [1,2,3,4,5,6,7,8,9,10]. HI of plasmid pAAV/HBV1.2 (containing a 1.2-fold overlength HBV genome) was found in a previous study to cause HBV replication and HBV surface antigen (HBsAg) expression that persisted for more than six months in approximately 40% of the injected mice [3]. It was speculated that the outcome after HI was also determined by the backbone of plasmids and the genetic background of injected mice. In our previous studies, we explored pAAV/HBV1.3 (containing a 1.3-fold overlength HBV genome) in C57BL/6 mice. We found that high levels of serum HBsAg and HBV DNA were detected at seven days post injection (dpi), and they declined to undetectable levels at 28 dpi, which indicated temporary HBV replication and antigen expression [2,7,11]. Therefore, we speculated that in addition to the backbone of plasmids and the genetic background of the injected specimen, viral characteristics could also be an independent determinant for viral replication and persistence in the HI mouse model.

Woodchuck hepatitis virus (WHV), like HBV, is a member of the family *Hepadnaviridae*. WHV and HBV share a remarkable similarity in genome organization and replication strategy [12]. Their nucleotide (nt) sequences were found to have a homology of 62%–70% [13]. Early studies revealed that the HBV capsid could encapsidate WHV polymerase-epsilon complexes and vice versa [14]. Likewise, defective HBV polymerase (POL) could be complemented by WHV counterparts, and vice versa [15]. Furthermore, HBV core antigen (HBcAg) and WHV core antigen (WHcAg) could interact with each other to produce chimeric capsids [15]. In our previous studies, we constructed two plasmids, pBS-HBV1.3 (pHBV1.3, containing a 1.3-fold overlength HBV genome) and pBS-WHV1.3 (pWHV1.3, containing a 1.3-fold overlength WHV genome) in pBluescript II SK(+) vector. After HI of pHBV1.3 in BALB/c mice, serum HBV antigen and HBV DNA peaked at seven dpi and normally disappeared at 28 dpi. However, we found that WHV viral DNA (vDNA) and antigens could persist up to 45 weeks after HI with pWHV1.3. The reason for WHV persistence in BALB/c mice was unknown and therefore, we wished to explore it.

In the present study, we constructed a series of chimeric WHV-HBV genomes based on pHBV1.3, in which different fragments of the HBV genome were substituted by the counterparts from WHV. We studied whether the chimeric WHV-HBV genomes would persist or be cleared in HI mice model and attempted to determine the viral genomic fragments that assisted in the persistence of the chimeric WHV-HBV genomes.

## 2. Materials and Methods

### 2.1. Ethics Statement

Female BALB/c mice (six to eight weeks old) used in this study were purchased from SJA Co., Ltd. (Changsha, China). Mice were kept in specific-pathogen-free (SPF) conditions with free access to water and foods. Guidelines for laboratory animal experiments were strictly followed. This study was conducted under the Permit Number 2010-361 from the Institutional Animal Care and Use Committee of Tongji Medical College (Wuhan, China).

### 2.2. Chimeric Woodchuck Hepatitis Virus and Hepatitis B Virus (WHV-HBV) Genomes

pWHV1.3 contained 1.3 copies of WHV genome (nt 1050–2190, GenBank J04514). pHBV1.3 contained 1.3 copies of the HBV genome (nt 1040–1986, GenBank AY220698). Both pWHV1.3 and pHBV1.3 were constructed based on the pBluescript II SK(+) vector. We constructed a series of chimeric genomes based on pHBV1.3 and pWHV1.3, in which the individual HBV sequences of pHBV1.3 were substituted by the corresponding WHV sequences.

**Figure 1:**
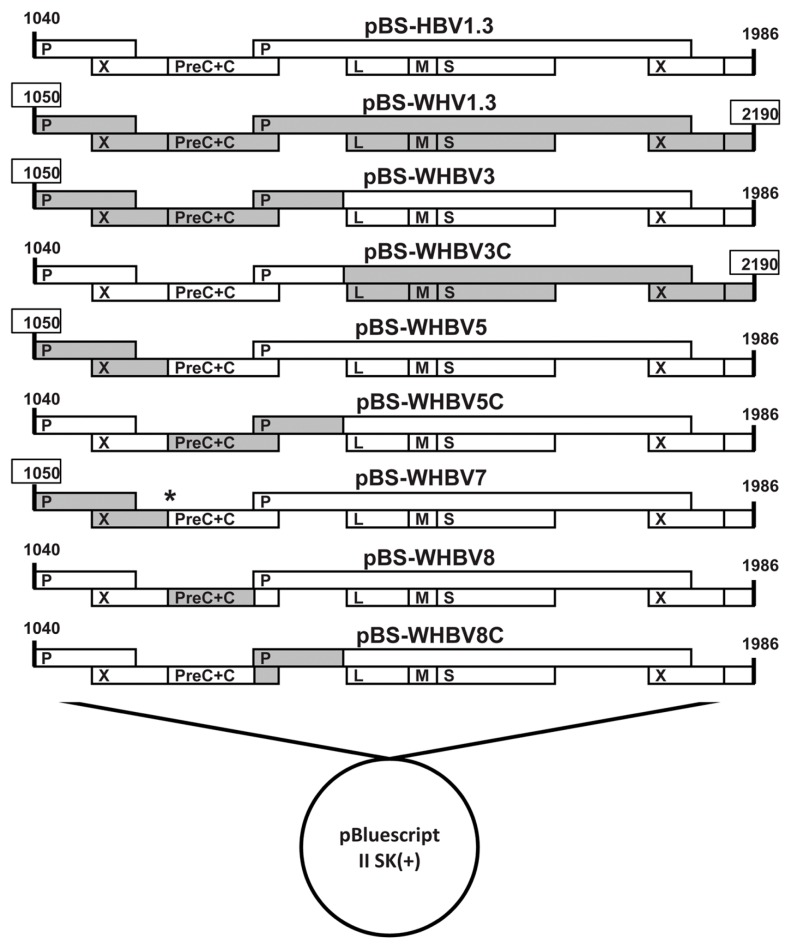
Recombinant woodchuck hepatitis virus-hepatitis B virus (WHV-HBV) genomes used in this study. pHBV1.3 (white) and pWHV1.3 (gray), which contained the 1.3-fold overlength genome of HBV and WHV, respectively, were both based on the pBluescript II SK(+) vector. The respective HBV genome sequences were substituted by the corresponding WHV sequences (gray bars), resulting in a series of chimeric WHV-HBV plasmids. *, the point mutation C1819T in the HBV preC region.

First, we constructed the chimeric plasmid pWHBV3, in which an HBV fragment (nt 1040-2817) was substituted by its counterpart WHV fragment (nt 1050–2950) (Figure 1, Figure S1 and Table S1). The WHV fragment (nt 1050–2950) was amplified from pWHV1.3 using high-fidelity polymerase chain reaction (PCR) with primers WHBV3F and WHBV3R (Table S2). Both pHBV1.3 and the cloned WHV fragment (nt 1050–2950) were double-digested with the *Kpn*I and *Bst*EII and then fused together. pWHBV3 contains the intact HBV *S* gene and WHV *C* gene, the chimeric *P* gene and two *X* genes.

Next, we constructed the chimeric plasmids of pWHBV5 and pWHBV5C, in which the inserted WHV fragment (nt 1050–2950) in pWHBV3 was subdivided. For pWHBV5, the WHV fragment (nt 1050–1933) replaced the corresponding HBV fragment (nt 1040–1818), based on pHBV1.3 (Figure 1, Figure S1 and Table S1). The WHV fragment (nt 1050–1933) was amplified from pWHV1.3 with primers WHBV4F and WHBV5FR, and the HBV fragment (nt 1819–2817) was amplified from pHBV1.3 with primers WHBV4R and WHBV5RF (Table S2). Then, the cloned WHV fragment (nt 1050–1933) and HBV fragment (nt 1819–2817) were fused together by overlapping PCR. The fused WHV-HBV fragment and pHBV1.3 were both digested with the restriction enzymes *Xho*I and *Bst*EII and then fused together, resulting in pWHBV5. pWHBV5C was constructed by the same strategy. For pWHBV5C, the HBV fragment (nt 1820–2817) was replaced by the corresponding WHV fragment (nt 1935–2950), based on pHBV1.3 (Figure 1, Figure S1 and Table S1). The HBV fragment (nt 1040–1819) was amplified from pHBV1.3 with primers WHBV5CF and WHBV5CFR, and the WHV fragment (nt 1935–2950) was amplified from pWHV1.3 using primers WHBV5CRF and WHBV5CR (Table S2). The cloned HBV fragment (nt 1040–1819) and WHV fragment (nt 1935–2950) were fused together by overlapping PCR. The fused HBV-WHV fragment and pHBV1.3 were both digested by the restriction enzymes *Pst*І and *Bst*EII and then fused together, resulting in pWHBV5C.

Finally, the chimeric plasmids of pWHBV8 and pWHBV8C were constructed based on pWHBV5C, in which the inserted WHV fragment (nt 1935–2950) in pWHBV5C was further subdivided (Figure 1, Figure S1, and Table S1). For pWHBV8, the HBV fragment (nt 1820–2331) was substituted by the corresponding WHV fragment (nt 1935–2449) based on pHBV1.3. The fragment (HBV nt 1040–1819, WHV nt 1935–2449) was digested from pWHBV5C by the restriction enzymes *Pst*Iand *Bsp*EI and then fused with the digested pHBV1.3 fragment using the same restriction enzymes, resulting in pWHBV8. For pWHBV8C, the HBV fragment (nt 2332–2817) was substituted by the corresponding WHV fragment (nt 2450–2950) based on pHBV1.3 (Figure 1, Figure S1 and Table S1). Two HBV fragments (nt 1040–2031 and nt 2035–2331) were amplified from pHBV1.3 using primers WHBV5CF/WHBV8CFR and WHBV8CRF/WHBV4R, respectively (Table S2), and were fused by overlapping PCR to change the redundant restriction enzyme *Bsp*EI site (TCT to AGC). The fused HBV fragment (HBV nt 1040–2031, AGC, HBV nt 2035–2331) and pWHBV5C were both digested with *Pst*I and *Bsp*EI and then fused together, resulting in pWHBV8C.

All of the chimeric HBV-WHV plasmids were sequenced by a commercial service (Beijing Genomics Institute, Shenzhen, China). We found that pWHBV5 had a stop codon at the beginning of the HBV preC region, leading to the termination of HBeAg production. Therefore, pWHBV7 was generated by site-directed mutagenesis using primers WHBV7FR and WHBV7RF to correct the second encoding codon from TAA to CAA in the HBV preC region (Table S2).

### 2.3. Hydrodynamic Injection (HI) of BALB/c Mice

HI procedures were performed as previously described [6]. Briefly, for each mouse, 10 μg of plasmids were diluted in normal saline (10% of mouse body weight) and injected via the tail vein within 8 s.

### 2.4. Serological Assays

Mouse sera were diluted (1:10), then enzyme-linked immunosorbent assay (ELISA; Kehua Tech, Xiamen, China) was used to measure viral antigens and related antibodies in sera (HBsAg, anti-HBs, HBeAg, anti-HBe, and anti-HBc). The results were indicated as optical density (OD) 450 nm, as measured on a microplate reader (cutoff = 0.1).

### 2.5. Measurementof Serum Viral DNA (vDNA)

At first, residual input DNA in sera was eliminated by DNase I (TaKaRa Bio Inc., Kusatsu, Japan). vDNA kits (Omega Bio-tek Inc., Norcross, GA, USA) were used to isolate DNA from viruses according to the manufacturer’s instructions. Primer QuantS and QuantAS (Table S3) were employed to measure vDNA via real-time PCR (qPCR) [16].

### 2.6. Measurementof WHV Core Antigen (WHcAg) and HBV Core Antigen (HBcAg) via Immunohistochemical Staining (IHC)

Mice were sacrificed and livers were collected. Then, livers were fixed with formalin and embedded in paraffin. WHcAg and HBcAg in paraffin sections were measured as previously described [17].

### 2.7. Measurement of Cytotoxic T Lymphocyte (CTL) Responses

Cytotoxic T lymphocyte (CTL) responses were measured using peptide stimulation and flow cytometry as previously described [18]. Briefly, at four weeks after HI with pHBV1.3 or pWHV-Sa (WHV) in C57BL/6 mice, the splenocytes were stimulated for six hours with the HBcAg peptide C93-100 (MGLKFRQL) or WHcAg peptide C13-21 (YQLLNFLPL; Sangon Biotech, Shanghai, China), respectively. For cell staining, anti-mIFN-γ-APC and anti-mCD8-PE (BD Biosciences, San Jose, CA, USA) were used. Dead cells were excluded using the LIVE/DEAD fixable dead cell stain kit (Invitrogen, Waltham, MA, USA). Cells were measured on FACSCalibur and analyzed using FlowJo software (Ashland, OR, USA).

### 2.8. Statistical Analysis

In this study, GraphPad Prism (GraphPad Software Inc., La Jolla, CA, USA) was applied to perform all of the statistical analyses. Student’s *t*-test was utilized to analyze any differences between the two independent groups. A statistical significance was set at *p* < 0.05. Results were indicated as means ± *SD*.

## 3. Results

### 3.1. WHV Could Persist in BALB/c Mice after HI

**Figure 2:**
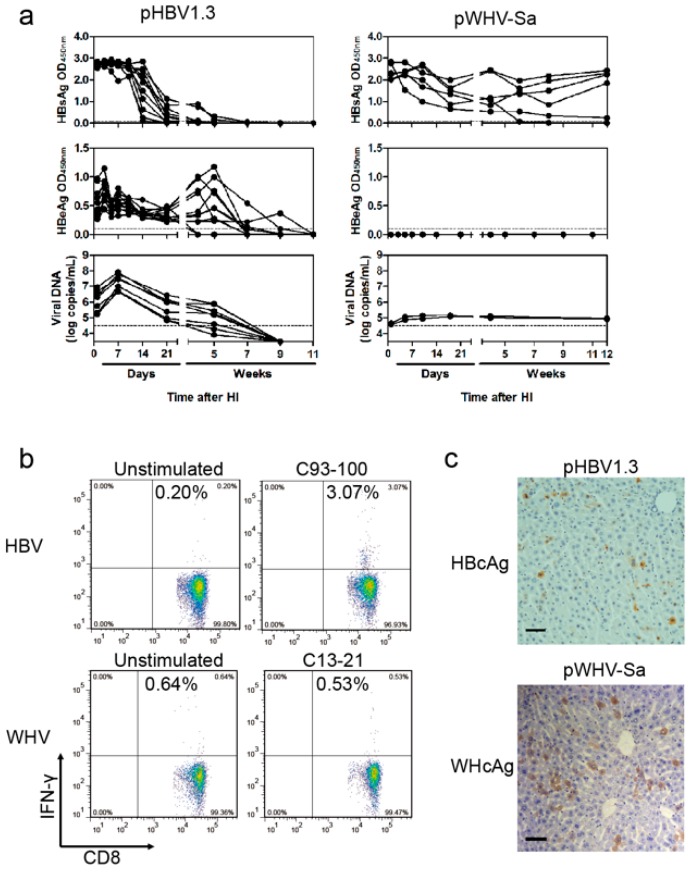
The kinetics of viral antigens and viral DNA (vDNA) in sera, the specific immune response, and hepatic antigen expression after HI with pHBV1.3 or pWHV-Sa in BALB/c mice. (**a**) In mouse sera, HBsAg and HBeAg were detected via ELISA, and the encapsidated vDNA was measured via real-time polymerase chain reaction (qPCR) at the indicated time points with pHBV1.3 or pWHV-Sa. The vector pBluescript II was used as negative control. The cutoff values for ELISA and qPCR were set as 0.1 and 3 × 104 copies/mL, respectively, which were indicated by the dotted line; (**b**) At four weeks after HI with pHBV1.3 (HBV) or pWHV-Sa (WHV) in C57BL/6 mice, the splenocytes were stimulated with HBV core antigen (HBcAg) peptide C93-100 or WHV core antigen (WHcAg) peptide C13-21, respectively. IFN-γ-producing CD8+ T cells were measured via flow cytometry; (**c**) At 10 days post infection (dpi), HBcAg or WHcAg expression in mouse livers were detected by immunohistochemical staining (IHC) after HI with pHBV1.3, pWHV-Sa or pBluescript II. Magnification: 200×.

We have explored HI of pHBV1.3 and pWHV1.3 in female BALB/c mice. After HI of pHBV1.3, HBsAg was detectable in the serum of all of the mice at one dpi. HBsAg expression levels peaked at seven dpi and began to decrease at 14 dpi. Then, serum HBsAg disappeared in nearly all mice at five weeks post infection (wpi). Serum HBeAg was detectable at one dpi, declined slowly and disappeared at nine wpi. HBV DNA in serum peaked at seven dpi and then decreased, finally disappearing at nine wpi (Figure 2a). Specific humoral immune responses were induced, as measured by specific viral antibodies (anti-HBs, anti-HBc, and anti-HBe), which was consistent with the kinetics of viral clearance (Figure S3).

After HI with pWHV1.3, we found that WHV vDNA and antigens could persist up to 45 weeks [19]. We constructed a chimeric WHV-HBV genome of pWHV-Sa, which contained the HBsAg a-determinant (amino acids (aa) 121–147) in place of the corresponding WHV sequence based on pWHV1.3 [19]. Similarly, the serum HBsAg expression by pWHV-Sa was highly positive from one dpi to 12 wpi after HI, though HBeAg was not produced by pWHV-Sa. Furthermore, the vDNA was maintained at a persistently low level in mouse serum until 12 wpi (Figure 2a). HI with the vector pBluescript II was used as negative control. At 10 dpi with pHBV1.3 or pWHV-Sa, HBcAg or WHV core antigen (WHcAg), respectively, were detectable by immunohistochemical staining (IHC) in mouse liver (Figure 2c). We compared the virus-specific CD8+ T cell responses induced in C57BL/6 mice at week 4 after HI with pHBV1.3 or pWHV-Sa. It was shown that HI with pHBV1.3 induced a stronger T cell response than with pWHV-Sa, which barely induced a specific immune response (Figure 2b). In this study, we tried to explore the characteristics of chimeric viruses that could promote the persistence of viral antigen.

### 3.2. Persistence of Chimeric WHV-HBV Genome of pWHBV3 in BALB/c Mice

To determine which region of the WHV genome is important for facilitating the persistence of chimeric virus, we first divided the 1.3-fold overlength WHV genome into two parts (WHV nt 1050–2950 and nt 2951–3323/0–2190). Then, we inserted the two WHV sequences into pHBV1.3, replacing the corresponding HBV sequences, to construct two chimeric genomes of pWHBV3 and pWHBV3C, respectively (Figure 1). In pWHBV3, the *X* gene, preC and *C* gene, and part of the *P* gene of HBV (nt 1040–2817) were substituted by the corresponding WHV genome (nt 1050–2950). The substitutions were carefully designed and the translation frames of viral proteins were unaffected. The *S* gene of HBV was kept intact in pWHBV3. In pWHBV3C, the *S* gene, part of the *P* gene and the entire *X* gene of HBV (nt 2818–3215/0–1986) were substituted by the corresponding WHV genome (nt 2954–3323/0–2190), but the HBV *C* gene was kept intact. To examine whether pWHBV3 and pWHBV3C were able to replicate and express antigens, Huh7 cells were transfected with pWHBV3 and pWHBV3C, respectively. ELISA assay of supernatants showed that pWHBV3 and pWHBV3C could produce high levels of HBsAg and HBeAg, respectively, at the indicated time points (Figure S2). pWHBV3 and pWHBV3C could replicate in Huh7 cells and had comparable replication levels (data not shown).

**Figure 3:**
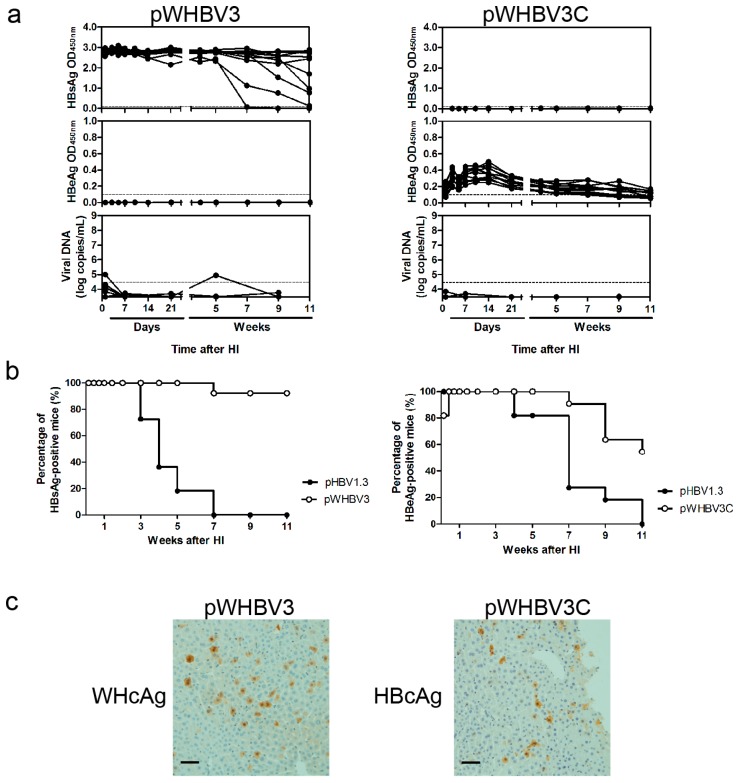
The kinetics of viral antigens and vDNA in sera, percentage of HBsAg and HBeAg antigenemia, and hepatic antigen expression after HI with pWHBV3 or pWHBV3C. (**a**) In mouse sera, viral antigens were measured via ELISA assay. The encapsidated vDNA was measured via qPCR. The cutoff values for ELISA and qPCR were set as 0.1 and 3 × 10^4^ copies/mL, respectively, which were indicated by the dotted line; (**b**) The percentages of HBsAg antigenemia in pWHBV3-injected mice and the percentage of HBeAg antigenemia in pWHBV3C-injected mice were compared with pHBV1.3-challenged mice at the indicated time points; (**c**) At 10 dpi, HBcAg and WHcAg in mouse liver was measured via IHC. Magnification: 200×.

We examined the replicative capacity of pWHBV3 and pWHBV3C in vivo by HI in mice. After HI with pWHBV3 in 13 BALB/c mice, high levels of HBsAg were produced in mouse sera from one dpi and remained positive in 92% of mice until 11 wpi (Figure 3a). However, only one mouse showed detectable serum vDNA at one dpi, which disappeared at seven dpi. The serum vDNA loads of other mice were very low and were under the detection limit of qPCR, which might be due to the fused POL encoded by the recombinant *P* gene. The POL of pWHBV3 was composed of WHV POL 1–175 aa and HBV POL 170–497 aa. However, HBeAg was detectable in 11 BALB/c mice after HI with pWHBV3C. At 11 wpi, approximately 50% of the mice showed persistent, low levels of HBeAg expression (Figure 3a). Similarly, pWHBV3C showed a weak replicative ability and serum vDNA was undetectable. At 10 dpi, HBcAg and WHcAg were distinctly detectable in mouse liver by IHC (Figure 3c). A specific humoral immune response, such as to anti-HBc, was undetectable in mice receiving pWHBV3C, though HBcAg was expressed in mouse livers (Figure S3). In mice that received HI with pWHBV3, the prolonged and high level of HBsAg antigenemia with undetectable levels of vDNA attracted our interest.

### 3.3. Persistent HBsAg Antigenemia after HI with Chimeric WHV-HBV Genomes of pWHBV5C and pWHBV8

We tried to define the shorter WHV region, which, replacing the homogenous HBV sequence, could support the persistent HBsAg antigenemia of the chimeric genomes in the mouse model. We divided the WHV sequence in pWHBV3 into two parts—nt 1050–1933 and nt 1935–2950—and then inserted them into pHBV1.3 to replace the corresponding HBV sequences, resulting in two chimeric plasmids. pWHBV5 contained the WHV fragment (nt 1050–1933), which primarily replaced the first X region (HBV nt 1040–1819) based on pHBV1.3, in which four open reading frames (ORFs) of the HBV genome were unaffected (Figure 1). pWHBV5C contained the inserted WHV fragment (nt 1935–2950), which replaced the corresponding HBV sequence (nt 1820–2817; Figure S1). The construction procedure of pWHBV5 caused a point mutation (C1819T) in the HBV genome, leading to a stop codon in the HBV preC region and to cessation of HBeAg expression. To eliminate the point mutation, pWHBV7 was generated by site-directed mutagenesis, in which the second encoding codon, TAA, was converted into CAA, so that HBeAg could be produced by pWHBV7. In vitro, both pWHBV7 and pWHBV5C could produce HBsAg in the supernatant of Huh7 cells after transfection. pWHBV7 also could produce HBeAg in the cellular supernatant (Figure S2). pWHBV5C and pWHBV7 showed comparable replication levels in Huh7 cells. We compared the characteristics of pWHBV7 and pWHBV5C after HI in BALB/c mice.

**Figure 4:**
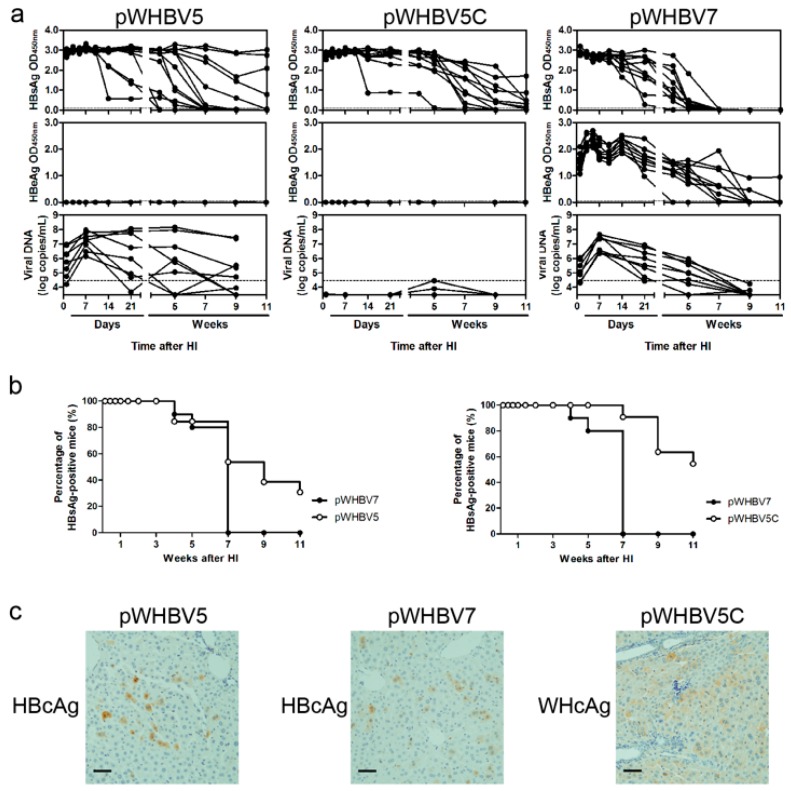
The kinetics of viral antigens and vDNA in sera, percentage of HBsAg antigenemia, and hepatic antigen expression after HI with pWHBV5, pWHBV5C or pWHBV7. (**a**) In mouse sera, viral antigens, and encapsidated vDNA were detected as described in Figure 3; (**b**) The percentages of HBsAg antigenemia in pWHBV5-, pWHBV5C-, and pWHBV7-challenged mice were calculated and compared at the indicated time points; (**c**) At 10 dpi, HBcAg and WHcAg expression in mouse liver was detected by IHC. Magnification: 200×.

In mice receiving pWHBV5C, HBsAg expression remained persistent in 54.5% of mice at 11 wpi (Figure 4). However, HBeAg and vDNA were undetectable in mouse serum. The low level of viral replication might be due to the recombinant POL in pWHBV5C. In mice receiving pWHBV7, HBsAg expression levels decreased and almost disappeared at five wpi. Accordingly, HBeAg expression gradually dropped and essentially disappeared at nine wpi. Serum vDNA peaked at seven dpi and disappeared at nine wpi, consistent with the kinetics of viral antigen (Figure 4a). At 10 dpi, HBcAg was expressed in approximately 7% of hepatocytes in mice receiving pWHBV7, while WHcAg was produced in approximately 2% of hepatocytes in mice receiving pWHBV5C (Figure 4c). Due to persistent HBsAg antigenemia and weak viral replication in pWHBV5C challenged mice, undetectable levels of antibodies were produced (Figure S4). In pWHBV7-challenged mice, antibodies, including anti-HBs and anti-HBe, were induced following the disappearance of antigen expression (Figure S4).

**Figure 5:**
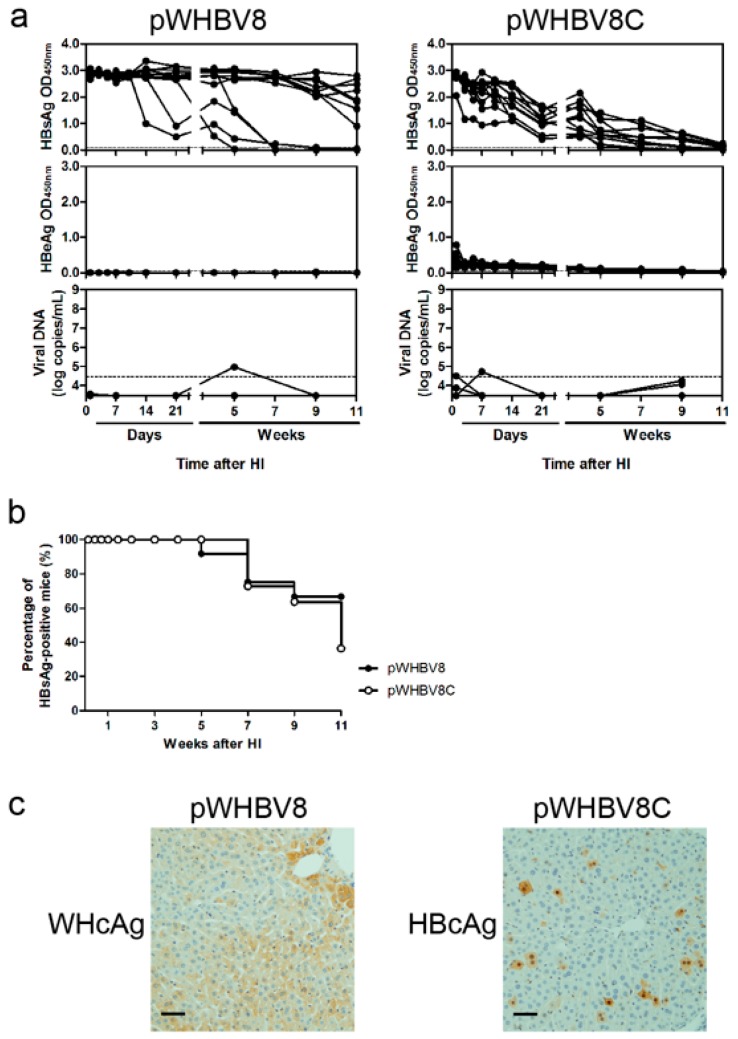
The kinetics of viral antigens and vDNA in sera, percentage of HBsAg antigenemia, and hepatic antigen expression after HI with pWHBV8 or pWHBV8C. (**a**) In mouse sera, viral antigens and encapsidated vDNA were detected as described in Figure 3; (**b**) The percentages of HBsAg antigenemia in pWHBV8- and pWHBV8C-injected mice were calculated at the indicated time points; (**c**) At 10 dpi, HBcAg and WHcAg expression in mouse liver was detected by ICS. Magnification: 200×.

We wondered whether the inserted WHV genome (nt 1935-2950) in pWHBV5C could be divided into smaller fragments that could be used to replace the corresponding HBV genome in pHBV1.3 to construct new chimeric plasmids leading to persistent HBsAg expression in mouse serum. Therefore, we further divided the inserted WHV genome in pWHBV5C into two parts—nt 1935–2449 and nt 2450–2950—to construct the chimeric plasmids pWHBV8 and pWHBV8C, using the start codon of HBV pre-genomic RNA (pgRNA; nt 2450). In pWHBV8, the HBV preC and C region fragments encoding the N-terminal 1–145 aa of HBcAg were replaced by the corresponding WHV sequences (nt 1935–2449), in which the HBV pgRNA was kept intact. In vitro, pWHBV8 could produce HBsAg, and pWHBV8C could produce high levels of HBsAg and HBeAg in the supernatant of Huh7 cells (Figure S2). We observed that HBsAg antigenemia persisted in 67% (8/12) of the mice receiving pWHBV8. In mice receiving pWHBV8C, HBsAg expression gradually reduced and then disappeared at 11 wpi. The serum HBeAg expression level was very low (Figure 5a). Replacing the HBV sequence encoding the C-terminal 150–183 aa of HBcAg with the corresponding WHV sequence in pWHBV8C might reduce HBeAg secretion. The serum vDNA loads were under the detection limit in mice receiving either pWHBV8 or pWHBV8C (Figure 5a). pWHBV8 persisted in 67% of mice at 11 wpi; however, pWHBV8C was cleared. At 10 dpi, WHcAg and HBcAg were expressed in the livers of mice that received pWHBV8 and pWHBV8C, respectively (Figure 5c). Anti-HBc antibody was induced in 18% (2/11) of mice receiving pWHBV8C. Finally, anti-HBs antibody was only produced in one mouse receiving pWHBV8 following HBsAg disappearance (Figure S4).

### 3.4. C1819T Mutation in pWHBV5 Led to HBsAg Persistence

Plasmid pWHBV5 is identical to pWHBV7, except that it contains a point mutation of C1819T in the HBV preC region. After HI in BALB/c mice, both pWHBV5- and pWHBV7-challenged mice could express HBsAg in serum; however, only mice that received pWHBV7 could produce serum HBeAg. In pWHBV5-challenged mice, serum HBsAg was produced at a high level and remained positive in 40% of mice at 11 wpi. However, in pWHBV7-challenged mice, serum HBsAg, HBeAg and vDNA simultaneously disappeared at seven wpi, indicating clearance of pWHBV7 (Figure 4a). The C1819T mutation in pWHBV5 makes the HBV initiator element (Inr) sequence more similar to the optimal Inr sequence (Figure S5), which could result in more effective transcription of pgRNA, a higher level of HBcAg production, and stronger viral replication than pWHBV7. We observed that HBcAg expression in liver at 10 dpi was stronger in pWHBV5-injected mice than in pWHBV7-injected mice (Figure 4a). Meanwhile, a more robust anti-HBc antibody response was detected in pWHBV5-injected mice due to the higher level of HBcAg expression (Figure S4). HBsAg persisted with the high titer of anti-HBc antibody in pWHBV5-injected mice.

## 4. Discussion

In this study, we created a series of chimeric WHV-HBV genomes based on a commonly used vector and explored hydrodynamic injection of the chimeric plasmids in BALB/c mice to study genomic factors that determine HBsAg persistence. Consistent with our previous results [19], a part of such constructs (pWHBV3, pWHBV5C, and pWHBV8) was able to persist in mice and continuously produce HBsAg. For example, pWHBV3 containing a WHV fragment (nt 1050–2950) supported persistent expression of HBsAg in 92% of challenged mice at 11 wpi, while pWHBV5C containing the shorter WHV fragment (nt 1935–2950) supported HBsAg persistence in only 54.5% of mice. Furthermore, pWHBV8 containing the smallest WHV fragment (nt 1935–2449) showed persistent HBsAg antigenemia in 67% of mice. Thus, we assume that the vector backbone does not represent a determinant for the persistence of WHV or the chimeric WHV-HBV genome in the HI mouse model. Compared with pHBV1.3, HBsAg expression in pWHV-Sa challenged mouse sera persisted until week 12 (Figure 2). We speculated that the low-level replication and the weak immune responses induced by pWHV-Sa might lead to persistent HBsAg expression. It is likely that the persistence is because the expression of viral antigens was below a critical level and did not provoke an effective host immune response before tolerance was established. pWHBV3-, pWHBV5C-, and pWHBV8-challenged mice consistently expressed intrahepatic WHcAg. Moreover, the complementary chimeric plasmids of pWHBV3C, pWHBV7, and pWHBV8C producing intrahepatic HBcAg could not support HBsAg persistence in BALB/c mice. It was reported that HBcAg played an important role in the clearance of HBV infection in the liver. Intrahepatic antiviral response may be triggered by HBcAg. In the HI mouse model, the intrahepatic antiviral response triggered by HBcAg is helpful not only for the clearance of infected hepatocytes but also for the input HBV DNA [3,4,20]. Our results showed that the mice receiving pWHBV3, pWHBV5C, or pWHBV8 with intrahepatic WHcAg expression provoked weak immune responses and failed to clear the chimeric plasmids in the liver, leading to HBsAg persistence. We speculated that WHcAg might present weaker antigenicity than HBcAg in BALB/c mice.

The C-terminal of HBcAg is rich in arginine residues and the C-terminal 39 residues of HBcAg are not required for capsid assembly, but function in pgRNA binding and vDNA synthesis [21]. It was reported that the last 10 residues of HBcAg played a key role in the immune response induction in the hydrodynamic mouse model [4]. The chimeric plasmid pWHBV8 encoded the recombinant core protein of the N-terminal 1–145 aa of WHcAg fused with the C-terminal 38 residues of HBcAg. We aligned the C-terminal 38 residues of HBcAg with WHcAg and found that this region was highly conserved. In our study, pWHBV8 persisted in mice with weak immune responses, though it contained only the C-terminal 10 residues of HBcAg. We speculated that the N-terminal 1–145 aa of HBcAg might be equally important for inducing strong immune responses. The weak immune response provoked by pWHBV8 might be due to the weak antigenicity of the assembled WHcAg particle by the N-terminal 1–145 aa of WHcAg in BALB/c mouse. Using the same strategy, we have constructed pWHV-HBV-Sa and pWHV-HBV-SaC145 based on pWHV1.3, in which WHV sequences were substituted by the HBV counterparts. In detail, pWHV-HBV-Sa contained HBsAg a-determinant (aa 121–147). On the other hand, pWHV-HBV-SaC145 was based on pWHV-HBV-Sa and contained one additional HBV fragment, which included the HBV preC region and the part of the C region encoding the N-terminal 1–145 aa of HBcAg. Of the BALB/c mice injected with pWHV-HBV-Sa, 83% of mice presented serum HBsAg positive at 12 wpi. However, the persistence rate of HBsAg antigenemia at 12 wpi in mice receiving pWHV-HBV-SaC145 was only approximately 33% (Figure S6). These results confirmed that in the HI BALB/c mouse model, the WHV genome fragment (nt 1935–2449) encoding the preC and N-terminal 145 aa of WHcAg could facilitate serum HBsAg persistence for the weak antigenicity of WHcAg. On the other hand, the HBV genome fragment (nt 1814–2331) encoding the preC and N-terminal 145 aa of HBcAg might promote the clearance of chimeric genomes and serum HBsAg in BALB/c mice. In the future, our approach may be used to further study the requirement of viral persistence or clearance in the mouse model based on the HI method.

Interestingly, the chimeric HBV-WHV genomes were able to stably express viral antigens in the liver. These constructs could be further used to express hepadnaviral antigens to study their pathogenic potential; for example, whether the persistent expression of HBsAg or HBeAg may impair intrahepatic immune responses to other antigens. Constructs with mutated viral antigens, such as secretion-defective HBsAg, may also be created to examine their impact on cellular processes.

It is still not clear how the persistent HBsAg and HBeAg expression was achieved in the HI mouse model. We showed that the replication-defective pWHV-Sa genome was not able to persistently express viral antigens under the same conditions [19], suggesting that viral replication is a prerequisite of continuous expression of viral antigens in the mouse liver. However, it is not yet possible to detect covalently closed circle DNA (cccDNA) in the mouse liver after HI due to the large quantity of input plasmids and replication intermediates. The copy numbers of cccDNA may be extremely low if their existence can even be confirmed. Thus, this will be an issue to be clarified in the future.

We speculated that HBeAg expression might act as an immune activator in BALB/c mice, especially in the innate immune response phase. Therefore, pWHBV7 with HBeAg expression could be cleared at seven wpi, though the viral replication level and the titer of anti-HBc were higher. Without HBeAg expression, HBsAg persisted for a longer time in pWHBV5-injected mice.

## Supplementary Materials

The following figures and tables are available online at www.mdpi.com/1999-4915/9/2/35/s1: Figure S1: The construction of recombinant WHV-HBV genomes; Figure S2: Viral antigens detected in Huh7 cells transfected with the chimeric WHV-HBV constructs; Figure S3: Antibody responses in pHBV1.3-, pWHBV3- and pWHBV3C-challenged mice; Figure S4: Antibody responses in pWHBV5-, pWHBV5C-, pWHBV7-, pWHBV8-, and pWHBV8C-challenged mice; Figure S5: Alignment of the basal core promoter (BCP) region and initiator (Inr) sequence of pHBV1.3 (HBV), pWHV1.3 (WHV), pWHBV3, pWHBV5C, pWHBV8, pWHBV5, and pWHBV7; Figure S6: HBsAg antigenemia in pWHV-HBV-Sa- and pWHV-HBV-SaC145-challenged mice; Table S1: The detailed composition of the chimeric WHV-HBV constructs; Table S2: Primers for chimeric WHV-HBV plasmids.
