# Supplementary material for: PreC and C Regions of Woodchuck Hepatitis Virus Facilitate Persistent Expression of Surface Antigen of Chimeric WHV-HBV Virus in the Hydrodynamic Injection BALB/c Mouse Model
Source: Viruses. 2017 Feb 21;9(2):35. doi: 10.3390/v9020035 (PMC5332954; doi:10.3390/v9020035)
Supplement: Supplementary file 1 [file viruses-09-00035-s001.pdf]

Supplementary Materials: PreC and C Regions of Woodchuck Hepatitis Virus Facilitate Persistent Expression of Surface Antigen of Chimeric WHV-HBV Virus in the Hydrodynamic Injection BALB/c Mouse Model

Weimin Wu, Yan Liu, Yong Lin, Danzhen Pan, Dongliang Yang, Mengji Lu and Yang Xu

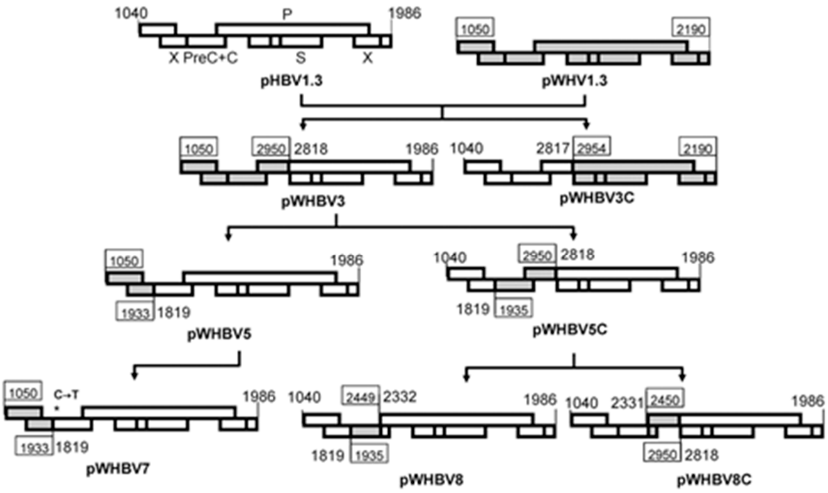

Figure S1. The construction of recombinant WHV-HBV genomes. pHBV1.3 (white) and pWHV1.3 (gray), which contained the 1.3-fold overlength genome of HBV and WHV, respectively. A series of chimeric WHV-HBV plasmids was constructed based on pHBV1.3 replaced with the corresponding WHV regions. The numbers indicated the nucleotide numbering of the HBV and WHV genomes. *: the point mutation C1819T in the HBV preC region.

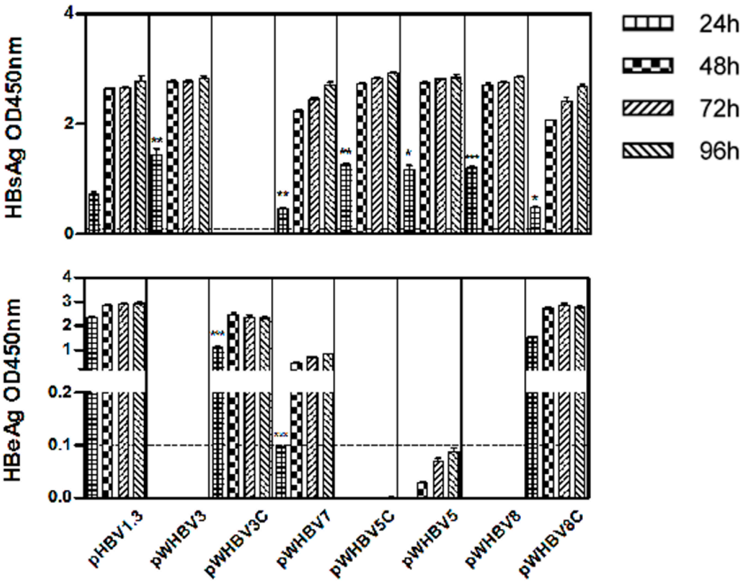

Figure S2. Viral antigens detected in Huh7 cells transfected with the chimeric WHV-HBV constructs. Huh7 cells were transiently transfected with the chimeric plasmids pWHBV3 (V3), pWHBV3C (V3C), pWHBV5 (V5), pWHBV5C (V5C), pWHBV7 (V7), pWHBV8 (V8), or pWHBV8C (V8C); pHBV1.3

(H1.3) and pWHV1.3 (W1.3) were used as controls. The expression levels of HBsAg and HBeAg in the supernatants were detected by ELISA at 24, 48, 72, and 96 hours after transfection. The cut off value was set as 0.1 and indicated by the dotted line. (*, significant, 0.01<p<0.05; **, very significant, 0.001<p<0.01; ***, extremely significant, p<0.001)

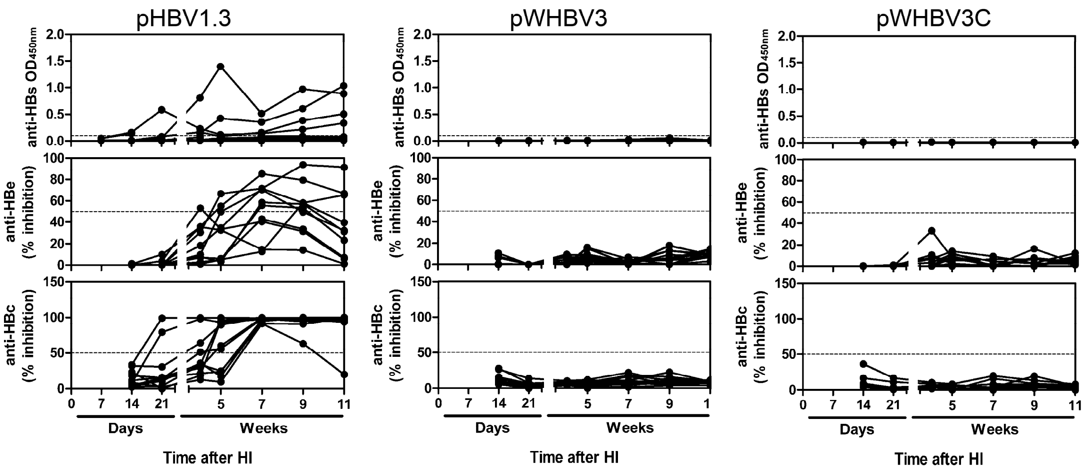

Figure S3. Antibody responses in pHBV1.3-, pWHBV3- and pWHBV3C-challenged mice. After HI with pHBV1.3, pWHBV3, or pWHBV3C, the humoral immune response was measured by ELISA with anti-HBs, anti-HBe, and anti-HBc antibodies at the indicated time points. The anti-HBe and anti-HBc antibodies were shown by the percentage of inhibition (% inhibition). The cut off value of anti-HBs antibody is 0.1. The cut off value of anti-HBe and anti-HBc antibodies is 50% inhibition and is indicated by the dotted lines.

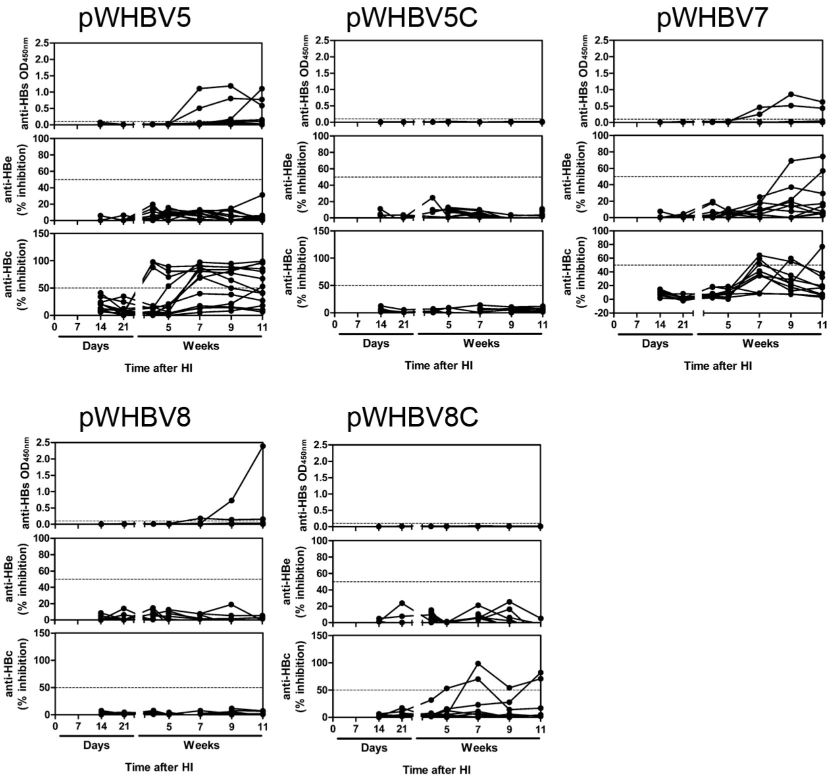

Figure S4. Antibody responses in pWHBV5-, pWHBV5C-, pWHBV7-, pWHBV8-, and pWHBV8C-challenged mice. After HI with pHBV5, pWHBV5C, pWHBV7, pWHBV8, or pWHBV8C, the humoral immune response was measured by ELISA with anti-HBs, anti-HBe, and anti-HBc antibodies at the

indicated time points. The anti-HBe and anti-HBc antibodies were shown by the percentage of inhibition (% inhibition). The cut off value of anti-HBs antibody is 0.1. The cut off value of anti-HBe and anti-HBc antibodies is 50% inhibition and is indicated by the dotted lines.

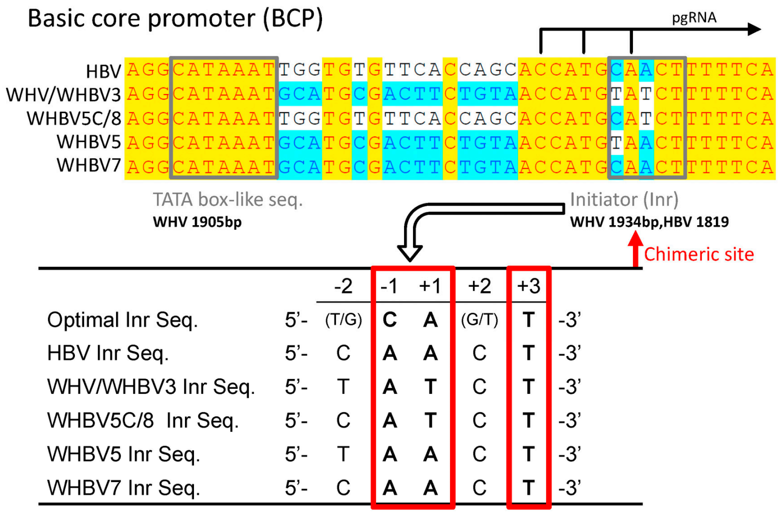

Figure S5. Alignment of the basal core promoter (BCP) region and initiator (Inr) sequence of pHBV1.3 (HBV), pWHV1.3 (WHV), pWHBV3, pWHBV5C, pWHBV8, pWHBV5, and pWHBV7.

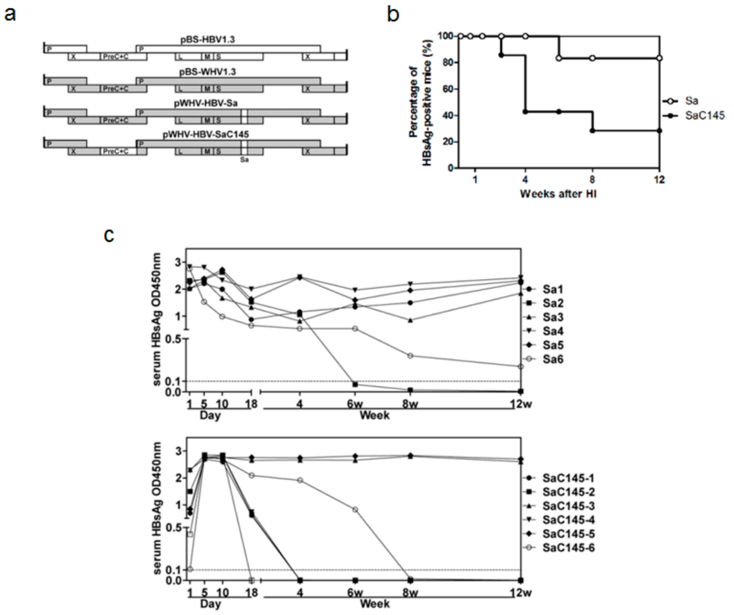

Figure S6. HBsAg antigenemia in pWHV-HBV-Sa- and pWHV-HBV-SaC145-challenged mice. (**a**) Schematic map of the chimeric WHV-HBV genomes of pWHV-HBV-Sa and pWHV-HBV-SaC145. (**b**) After HI with pWHV-HBV-Sa or pWHV-HBV-SaC145 in BALB/c mice, the percentage of HBsAg antigenemia was measured at the indicated time points. (**c**) HBsAg expression in serum was detected by ELISA after HI with pWHV-HBV-Sa or pWHV-HBV-SaC145 at the indicated time points. The cut off value is 0.1 and is indicated by the dotted lines.

Table S1. The detailed composition of the chimeric WHV-HBV constructs.

Plasmid name	Composition
pBS-HBV1.3	HBV nt1040–3215/0-1986
pBS-WHV1.3	WHV nt1050-3323/0-2190
pWHBV3	WHV nt1050–2950 + HBV nt2818-3215/0-1986
pWHBV5	WHV nt1050-1933 + HBV nt1819-1986
pWHBV5C	HBV nt1040-1819 + WHV nt1935-2950 + HBV nt2818-1986
pWHBV8	HBV nt1040-1819 + WHV nt1935-2449 + HBV nt2332-1986
pWHBV8C	HBV nt1040-2031 + AGC + HBV nt2035-2331 + WHV nt2450-2950 + HBV nt2818-1986

The numbering of the HBV genome is according to the Genbank accession NO. AY220698. The numbering of the WHV genome is according to the Genbank accession NO. J04514.

Table S2. Primers used for the construction of the chimeric WHV-HBV plasmids.

Designation	Polarity	Sequence
WHBV3F	Sense	GGCGGTACCCACATGTTAAGAAAAT
WHBV3R	Antisense	GGCGGTCACCCTTTAAAAGTCAAAGT
WHBV4F	Sense	ACGCTCGAGGCTGGGTACCACATGTTAAG
WHBV4R	Antisense	TATGGTGACCCGCAAAATGAGGCGCTAC
WHBV5FR	Antisense	TGAAAAAGTTACATGGTTACAGAAGTCGCATGCA
WHBV5RF	Sense	TGTAACCATGTAACTTTTTCACCTCTGCCTAATCATC
WHBV5CF	Sense	TGCAACTGCAGTGGATATCCTGCTTTAATG
WHBV5CR	Antisense	GGCGGTGACCCTTTAAAAGTCAAAGT
WHBV5CFR	Antisense	TGAAAAAGATGCATGGTGCTGGTGAAC
WHBV5CRF	Sense	CACCATGCATCTTTTTCACCTCTGCC
WHBV7FR	Antisense	TGAAAAAGTTGCATGGTTACAGAAGTCGCATGCA
WHBV7RF	Sense	TGTAACCATGCAACTTTTTCACCTCTGCCTAATCATC
WHBV8CRF	Sense	TTAGAGAGCCCGGAACATTGTTCACC
WHBV8CFR	Antisense	CCGGGCTCTCTAAGGCCTCC

Table S3. Primers used for the real-time PCR to detect serum viral DNA after HI.

Designation	Polarity	Sequence
QuantS	Sense	TGCCTCATCTTCTTGTTGGTTCT
QuantAS	Antisense	CCCCAAAACCAAATCATCCATATA

© 2017 by the authors. Licensee MDPI, Basel, Swizerland. This article is an open access article distributed under the terms and conditions of the Creative Commons Attribution (CC-BY) license (http://creativecommons.org/licenses/by/4.0/).
